# Oxygen uptake efficiency plateau is unaffected by fitness level - the NOODLE study

**DOI:** 10.1186/s13102-024-00939-w

**Published:** 2024-07-10

**Authors:** Przemysław Kasiak, Tomasz Kowalski, Kinga Rębiś, Andrzej Klusiewicz, Dorota Sadowska, Adrian Wilk, Szczepan Wiecha, Marcin Barylski, Adam Rafał Poliwczak, Piotr Wierzbiński, Artur Mamcarz, Daniel Śliż

**Affiliations:** 1 3rd Department of Internal Medicine and Cardiology, Medical University of Warsaw, Warsaw, Poland; 2 Doctoral School, Medical University of Warsaw, Warsaw, Poland; 3 Department of Physiology, Institute of Sport - National Research Institute, Warsaw, Poland; 4 Faculty of Physical Education and Health, Branch in Biala Podlaska, Jozef Pilsudski University of Physical Education in Warsaw, Biała Podlaska, Poland; 5 Department of Internal Medicine, Rehabilitation and Physical Medicine, Medical University of Lodz, Łódź, Poland

**Keywords:** Oxygen uptake efficiency plateau, Cardiopulmonary exercise testing, Endurance athletes, Prediction equation, Cardiorespiratory fitness, Cardiovascular health

## Abstract

**Background:**

Endurance athletes (EA) are an emerging population of focus for cardiovascular health. The oxygen uptake efficiency plateau (OUEP) is the levelling-off period of ratio between oxygen uptake (VO_2_) and ventilation (VE). In the cohort of EA, we externally validated prediction models for OUEP and derived with internal validation a new equation.

**Methods:**

140 EA underwent a medical assessment and maximal cycling cardiopulmonary exercise test. Participants were 55% male (*N* = 77, age = 21.4 ± 4.8 years, BMI = 22.6 ± 1.7 kg·m^− 2^, peak VO_2_ = 4.40 ± 0.64 L·min^− 1^) and 45% female (*N* = 63, age = 23.4 ± 4.3 years, BMI = 22.1 ± 1.6 kg·m^− 2^, peak VO_2_ = 3.21 ± 0.48 L·min^− 1^). OUEP was defined as the highest 90-second continuous value of the ratio between VO_2_ and VE. We used the multivariable stepwise linear regression to develop a new prediction equation for OUEP.

**Results:**

OUEP was 44.2 ± 4.2 mL·L^− 1^ and 41.0 ± 4.8 mL·L^− 1^ for males and females, respectively. In external validation, OUEP was comparable to directly measured and did not differ significantly. The prediction error for males was − 0.42 mL·L^− 1^ (0.94%, *p* = 0.39), and for females was + 0.33 mL·L^− 1^ (0.81%, *p* = 0.59). The developed new prediction equation was: 61.37–0.12·height (in cm) + 5.08 (for males). The developed model outperformed the previous. However, the equation explained up to 12.9% of the variance (*R* = 0.377, R^2^ = 0.129, RMSE = 4.39 mL·L^− 1^).

**Conclusion:**

OUEP is a stable and transferable cardiorespiratory index. OUEP is minimally affected by fitness level and demographic factors. The predicted OUEP provided promising but limited accuracy among EA. The derived new model is tailored for EA. OUEP could be used to stratify the cardiorespiratory response to exercise and guide training.

**Supplementary Information:**

The online version contains supplementary material available at 10.1186/s13102-024-00939-w.

## Introduction

Endurance athletes (EA) regularly participate in competitions and are exposed to high physical loads [1]. Cardiovascular diseases (CVD) are still a significant problem in the health care system [2]. Strenuous exercise could elevate the risk of CVD [3]. Hence, the development and validation of novel, reliable indices remain crucial to enable a comprehensive interpretation of cardiorespiratory fitness [4]. The gold-standard metric of athletic performance is oxygen uptake (VO_2_) [5]. However, VO_2_ is not the only one, and the usefulness of the other measures should be investigated [6, 7].

EA usually does not fit into the cardiorespiratory reference values from general population [8–10]. Both, the VO_2_ and ventilation (VE) are poorly predicted and mostly underestimated by common prediction equations in EA [8, 11]. Moreover, measurements of absolute value of variables are often an insufficient source of knowledge about cardiovascular physiology in EA [12, 13]. Recent focus has been applied to ratios of oxygen uptake efficiency measures which is described by the correspondence between VO_2_ and VE [14, 15]. Moreover, it is sometimes not feasible to perform cardiopulmonary exercise test (CPET) and directly measure cardiorespiratory fitness. Thus, the importance of prediction equations based on non-exercise and body measures emerge [16].

The oxygen uptake efficiency plateau (OUEP) was originally introduced by Sun et al. [17]. OUEP relates to different periods of exercise than the oxygen uptake plateau [17, 18]. OUEP explains the levelling-off between VO_2_ and VE [17]. OUEP can be plotted in the majority of exercise tests because it occurs early, just before the aerobic threshold [17, 19]. OUEP occurs before hyperventilation due to demanding exercise leads to acidemia [17]. If OUEP falls below 65% of the predicted value, there is a suspected pathology [19]. However, among numerous cardiorespiratory indices, EA often noted an underestimation when compared to the untrained subjects [20, 21]. As it merges cardiac and respiratory systems, it may be superior to previous risk indicators (i.e. heart rate, ventilatory efficiency, oxygen pulse, etc.) [19].

The issues of prevention and diagnosis of cardiovascular diseases (CVD) among EA are increasingly gaining attention [3]. Adjustment between VE and VO_2_ emerge as a valuable, interesting direction in sports cardiology. Hypo- or hyper- ventilation is influenced mostly by cardiovascular functions, however, peripheral and pulmonary factors also contribute here [22]. Merge between VE and VO_2_ could be used in clinical setting to grade the CVD and in the sports cardiology to stratify impairment in physical training or to assess fitness [23–25].

OUEP should be stable even in highly fit athletes and varies only slightly [17]. However, no studies have confirmed its replicability in the EA population so far. Moreover, the prediction equation for OUEP has never been externally validated on other populations. We noticed a significant understudied area of knowledge. This research corresponds and complements to the previous NOODLE studies about ventilatory efficiency and oxygen uptake efficiency slope in EA [10, 26].

In this research, we aimed to: (1) clarify whether OUEP remains reproducible in a group of EA by external validation of the previous prediction equation, and (2) systemize the usefulness of OUEP in EA by development and internal validation of a new non-exercise model.

## Materials and methods

### Study setting

This study received approval from the Bioethics Committee of the Medical University of Warsaw. Participants provided their written informed consent. We applied the STROBE statement of EQUATOR Network guidelines [27]. The checklist is included in the Supplementary Material 1 (Table S1). The recruitment period was 2022–2023.

### Eligibility criteria

Firstly, we applied the following inclusion criteria for EA: (1) age ≥ 18 years, (2) ≥ 4-year experience in regular endurance training, (3) membership in a sports association and national elite or development teams, and (4) regular participation in competitions on the regional and international levels. Participants were assigned to Class 3–5 in McKay classification framework [28].

Further, we ensured a consultation with a medical doctor to confirm the overall health of our participants. We used rigorous exclusion criteria that considered past medical history and ongoing symptoms. The physician looked for the presence of any of the following: pulmonary diseases, CVD, neurological and mental disorders, haematological deviations, and orthopaedic injuries; and we asked about habitual tobacco smoking. If we confirmed a past medical history, the physician refused the subject from CPET. Precise definitions of examined abnormal health criteria are described in Table 1.

**Table 1:** Abnormal health findings considered as the exclusion criteria

**1. Pulmonary diseases**
- *Chronic obstructive pulmonary disease* - poorly controlled bronchial asthma - blood saturation < 95%
**2. Cardiovascular diseases**
- significant heart rhythm disturbances in the 12-lead ECG (e.g., ventricular and supraventricular arrhythmias, atrial fibrillation) - features of myocardial ischemia, - prolongation of the QT interval in the 12-lead ECG - structural heart disorders detected in cardiac echocardiography (e.g., hemodynamically relevant valvular defects, hypertrophic cardiomyopathy, systolic dysfunction of the right or left ventricle), - decompensated blood pressure (with increases above 160/100 mmHg).
**3. Neurological and mental disorders**
**4. Significant deviations found in CBC**
- Leukocytosis above 10 000 ∙ mm^− 3^ - Anaemia with Hb level < 10 g ∙ d^− 1^
**5. Exercise-limiting musculoskeletal injuries**

*Note* Any of the above health conditions were considered as the mandatory exclusion criteria during pre-participation medical follow-up. Abbreviations: 12-lead ECG, 12-lead electrocardiography; CBC, complete blood count; Hb, blood haemoglobin concentration

**Table 2:** Study population

Variable		All EA [*N* = 140]	Males [*N* = 77]	Females [*N* = 63]
A. Baseline characteristics				
Age (years)		22.7 ± 4.6	21.8 ± 4.8	23.8 ± 4.2
Height (cm)		174.8 ± 9.9	181.6 ± 6.3	166.3 ± 6.2
Body weight (kg)		69.3 ± 10.1	76.1 ± 7.6	61.0 ± 5.5
BMI (kg·m^− 2^)		22.6 ± 1.7	23.1 ± 1.7	22.1 ± 1.6
BSA (m^2^)		1.84 ± 0.12	1.97 ± 0.18	1.68 ± 0.10
Sport discipline	Triathlon or cycling	56 (40.0)	30 (47.6)	26 (33.8)
	Speedskating	59 (42.1)	26 (41.3)	33 (42.9)
	Other endurance sports	25 (17.9)	7 (11.1)	18 (23.3)
B. Exercise performance				
HR (beats·min^− 1^)		190.9 ± 8.6	190.8 ± 8.7	191.0 ± 9.1
VE (L·min^− 1^)		154.5 ± 34.1	176.3 ± 26.3	127.8 ± 21.1
VO_2_peak (L·min^− 1^)		3.86 ± 0.82	4.40 ± 0.64	3.21 ± 0.48
VO_2_peak/kg (mL·kg^− 1^·min^− 1^)		55.2 ± 8.6	57.8 ± 9.0	52.1 ± 7.0
% pred. VO_2_peak		144.5 ± 25.9	130.6 ± 20.2	161.4 ± 21.8
OUES (mL·min^− 1^/L·min^− 1^)		3.96 ± 0.90	4.41 ± 0.87	3.41 ± 0.58
OUEP (mL·L^− 1^)		42.7 ± 4.7	44.2 ± 4.2	41.0 ± 4.8

*Abbreviations* BMI, body mass index; BSA, body surface area; HR, peak heart rate; VE, peak minute ventilation; VO_2_peak, peak oxygen uptake; OUES, oxygen uptake efficiency slope; OUEP, oxygen uptake efficiency plateau

*Note* Upper rows (Part A) present characteristics of study group and lower rows (Part B) present exercise performance. OUES was calculated from VO_2_/VE_log_ during the whole exercise effort. OUEP was considered the highest continuous 90-second average from the VO_2_/VE ratio. Measures are presented as mean ± standard deviation or number (percentage). Predicted VO_2_peak was calculated from the Wassermann and Hansen Eq. (31)

Finally, we considered effort as maximal when there was a: (1) ≥ 30-s VO_2_ plateau, (2) respiratory exchange ratio (RER) ≥ 1.05, (3) maximal heart rate ≥ 80% of the age-predicted, (4) EA declined further exercises, and (5) declared exhaustion was ≥ 18 points on the Borg scale. We chose these criteria from cardiopulmonary reference data for endurance athletes [8, 29]. All listed criteria of maximal effort were obligatory to include the EA in this study. If the CPET was submaximal the participant was not included in the analysis. The CPET was defined as submaximal when EA did not reach all of the previously listed criteria. Failure to meet any of these criteria excluded the participant.

**Fig. 1:**
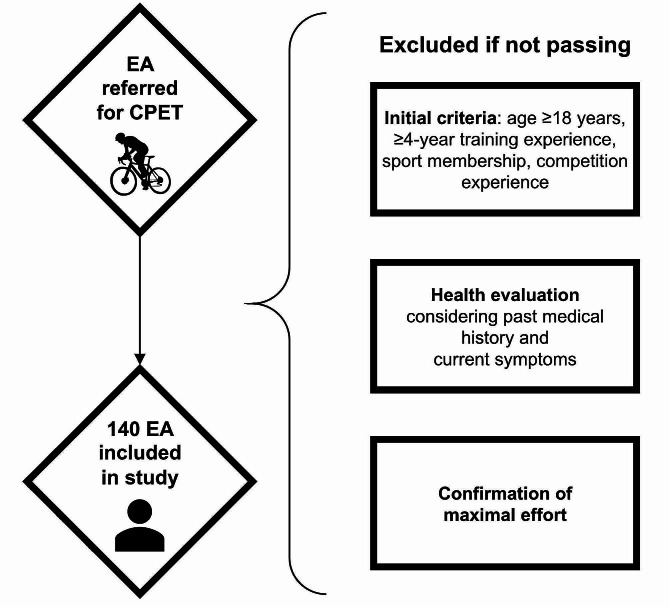
Schematic representation of the participants recruitment procedure. *Abbreviations* EA, endurance athlete; CPET, cardiopulmonary exercise test

The flowchart presenting recruitment process is presented on the Fig. 1.

### Cardiopulmonary exercise testing

Continuous ramp CPET was performed on a Cyclus II Cycling Ergometer (RBM, Leipzig, Germany). All CPET was conducted in unified procedures of the Institute of Sport - National Research Institute in Warsaw (https://insp.pl, accessed on 6th March 2024). The exercise began with pedalling without load for 2–3 min to warm up. The incremental protocol started with a workload between 55 and 70 W and increased by 0.17–0.28 W·s^− 1^. The starting workload and grading of workload during CPET were set after reaching agreement between the supervising physiologist and each single EA in the aforementioned ranges. Participants were guided by the physiologist and encouraged verbally to achieve maximum results.

### Measuring procedures

We obtained several body indices: height, body weight, BMI, and body surface area (BSA). We measured body weight with the TANITA scale (TANITA Corporation, Arlington Heights, IL, USA) before breakfast. We measured height using the stadiometer (Seca GmbH & Co., Hamburg, Deutschland) in the morning (along with body weight). We calculated BSA from Du Bois & Du Bois Eq. [49]. Raw breath-by-breath data for ventilatory measures were collected using the Hans Rudolph V2 Face Mask (Hans Rudolph, Inc, Shawnee, KS, USA). During the data collection process, we used the Cortex B3 Metamax metabolic system (CORTEX Biophysik GmbH, Leipzig, Germany). We recorded: VE, VO_2_, VCO_2_, respiratory rate, and tidal volume. Gas analysing devices were calibrated individually for all participants before CPET. A Polar H10 heart rate sensor with a chest strap was used to measure heart rate. The heart rate sensor was continuously paired with the Cyclus II Cycling Ergometer. All obtained indices were averaged in the 15-second intervals. We excluded the first minute of loaded protocol to minimize noise variables and determined OUEP as the highest continuous 90-second average of VO_2_/VE ratio plotted against time [17]. We excluded all the enrolled individuals with missing data to ensure the maximal credibility of the results.

### Screening for prediction equation for OUEP

To select previous prediction equations for OUEP, we examined the 5 databases: PubMed, Scopus, Web of Science, Google Scholar, and Medline. Applied keywords were: “OUEP”, “oxygen uptake efficiency plateau”, “cardiopulmonary exercise tests”, “prediction equation”, “reference values”, and “oxygen uptake efficiency”. We included only models which were derived from healthy, adult populations (age ≥ 18 years, no existing co-morbidities). One prediction equation for OUEP has been found [17]:

$$\eqalign{{\rm{OUEP}}{\mkern 1mu} {\rm{[mL}} \cdot {{\rm{L}}^{ - 1}}{\rm{]}} =& {\mkern 1mu} 42.18 -   0.189 \cdot {\rm{age}}{\mkern 1mu} \left[ {in\,years} \right] \, & \cr & + 0.036 \cdot {\rm{height}}{\mkern 1mu} \left[ {in\,cm} \right]{\mkern 1mu} \left] { - 3.02} \right[if\,female{\rm{]}} \cr}$$

The equation was derived from the mixed treadmill and/or cycling protocol from a healthy population of 417 participants aged 17–74 years. The population of the derivation study also had well-trained athletes (*N* = 57) with > 140% of predicted VO_2_peak according to the Wasserman & Hansen Eq. (31) and those well-trained athletes were not included in the model derivation process. The CPET started with a 3-min resting and 3-min warm-up followed by an incremental ramp cycling protocol and terminated with at least 2-min recovery.

### Statistical analysis

To determine the data distribution, we used the Shapiro-Wilk test and visually examined the corresponding Q-Q plots. We presented categorical variables as number (percentage) and continuous variables as mean ± standard deviation. We used IBM SPSS (version 29.0, IBM, Armonk, NY, USA) for analyses and GraphPad Prism (version 10.1, GraphPad Software, San Diego, California USA) to develop the plots. We set *p* < 0.05 as significant.

The external validation of the prediction equation for OUEP was determined by comparing observed and predicted values by Student’s t-test for independent samples and calculating root-mean-square error (RMSE). The compliance of predicted to observed OUEP was shown in Bland-Altman plots. The variance explained by the previous model was examined by regressing observed OUEP against predicted OUEP and presented as an adjusted coefficient of determination (R^2^). The correlation of OUEP and VO_2_peak was assessed by the Pearson Correlation Coefficient (R). The new model was derived with the stepwise multiple linear regression. The method for model derivation was selected by assessment of data assumptions (collinearity, correlations, independence of observations, residuals, and leverage plots). Especially, we included only significant variables with *p* < 0.05. Finally, the model was internally validated with the bootstrapping from 10,000 iterations [30].

The sample size was evaluated post-hoc in the G*Power software [31]. For all applied statistical methods, the study population achieved significance, a large effect size, and a power of 0.99. Results were presented following the current 11th Edition of guidelines of the American Medical Association Manual of Style: A Guide for Authors and Editors [32].

## Results

### Study population

Of 140 EA, 77 (55.0%) were male and 63 (45.0%) were female. Table 2 presents the brief participants’ basic demographic and exercise characteristics, while a detailed description of the study population is provided in the Supplementary Material (Table S2). Participants represented the following endurance disciplines: 56 (40.0%) triathlon or cycling, 59 (42.1%) speedskating, and 25 (17.9%) other disciplines. The predicted VO_2_peak was 144.5 ± 25.9% and ranged from 90.6 to 216.2% according to Wasserman and Hansen equation. Females had lower OUEP than males for an average of 3.2 mL·L^− 1^ (*p* < 0.001). OUEP was 44.2 ± 4.2 mL·L^− 1^ (range 36.2–54.2 mL·L^− 1^) and 41.0 ± 4.8 mL·L^− 1^ (range 29.4–53.0 mL·L^− 1^) for males and females, respectively. VO_2_peak was significantly correlated with OUEP (*R* = 0.32, *p* < 0.001).

### Derivation of the new model

We evaluated several non-exercise measures (sex, age, height, body weight, BMI, and BSA) for their suitability in building the model. The parsimonious bivariable model included the height and was adjusted to sex. The derived equation for OUEP is presented in Table 3.

**Table 3:** Multivariable prediction equation for OUEP

Covariate	Estimate	Standard Error	β	95% CI		*p*-value
				LL	UL	
Intercept	61.369	9.92	---	41.747	80.991	< 0.001
Sex	5.078	1.18	0.539	2.749	7.407	< 0.001
Height	–0.123	0.06	–0.257	–0.240	–0.005	0.041

*Abbreviations* 95% CI, 95% confidence interval; LL, lower limit; UL, upper limit

*Note* Sex is 1 for males and 0 for females

**Fig. 2:**
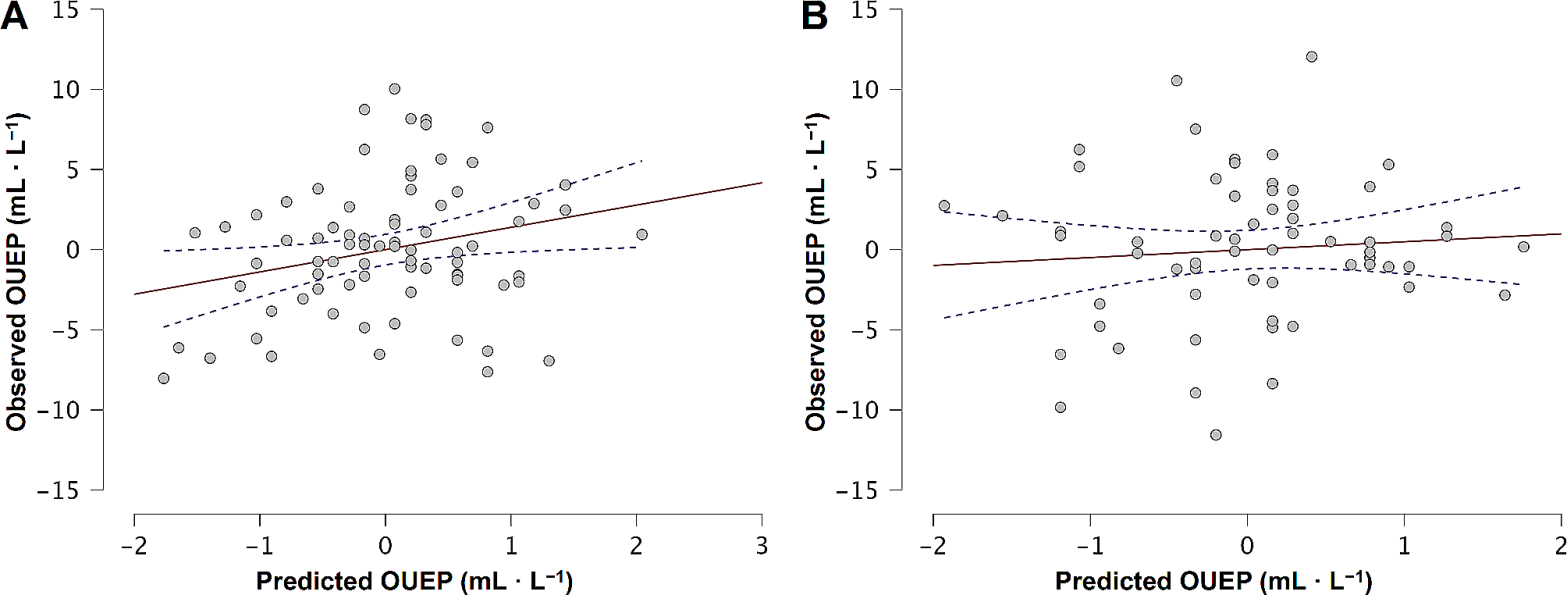
Correlation between observed and predicted OUEP. *Abbreviations* OUEP, oxygen uptake efficiency plateau. *Note* Panel A represents males and panel B represents females. The central red line represents the trend. The blue dotted lines represent 95% confidence intervals. The plot presents univariable regression analysis of observed OUEP against predicted OUEP

**Fig. 3:**
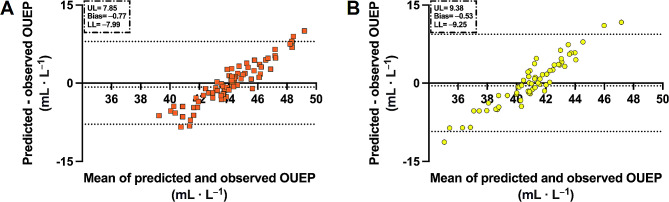
Bland-Altman plots of the prediction accuracy of derived models. *Abbreviations* UL, upper limit of agreement; LL, lower limit of agreement. *Note* Panel A (orange color) represents males and panel B (yellow color) represents females. Upper dotted line represents upper limit of agreement and lower dotted line represent lower limit of agreement. Area between dotted lines represent model’s accuracy

The model was responsible for 12.9% of the variance in OUEP (*R* = 0.377, R^2^ = 0.129). Overall regression was significant (F(2, 137) = 11.33, *p* < 0.001). In Fig. 2 we compared observed and predicted OUEP by regressing one against another. As expected by the limited R^2^ the data were scattered both for males and females. The model’s RMSE was 4.39 mL·L^− 1^. Predicted OUEP equals 42.67 mL·L^− 1^ which was 100.2% of the observed values. The difference was 0.07 mL·L^− 1^ for the whole population. Bland-Altman plots presenting the agreement between observed and predicted OUEP are in Fig. 3. In both males and females, the OUEP was slightly underestimated. The bias was − 0.77 mL·L^− 1^ and − 0.53 mL·L^− 1^ for males and females, respectively. The limit of agreement was wider in females (–9.25 mL·L^− 1^ to 9.38 mL·L^− 1^) than in males (–7.99 mL·L^− 1^ to 7.85 mL·L^− 1^).

### External validation of prediction equation for OUEP

The external model overestimated OUEP by only 0.08 mL·L^− 1^. The total difference was 0.19% and RMSE was 4.48 mL·L^− 1^. In the whole population, values did not differ significantly (t(278)= − 0.18, *p* = 0.86). The external model contributed to the 9.9% of the variance in the directly observed OUEP (R^2^ = 0.099).

**Fig. 4:**
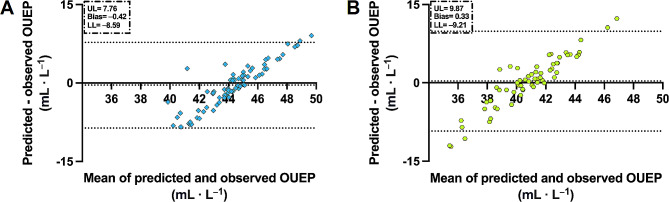
Bland-Altman plots for external validation of previous models. *Abbreviations* UL, upper limit of agreement; LL, lower limit of agreement. *Note* Panel A (blue color) represents males and panel B (green color) represents females. Upper dotted line represents upper limit of agreement and lower dotted line represent lower limit of agreement. Area between dotted lines represent model’s accuracy

Briefly, higher bias was noted for males than females. For males, the OUEP was underestimated by 0.42 mL·L^− 1^ (0.94%). As in the total population, predicted values also did not differ significantly in males (t(152)= − 0.86, *p* = 0.39). RMSE for males was 4.16 mL·L^− 1^. A similar relationship was observed among females. However, the model overestimated OUEP by 0.33 mL·L^− 1^ (0.81%) in females. The error was not significant (t(124) = 0.54, *p* = 0.59). RMSE for females was 4.84 mL·L^− 1^. A visual representation of model prediction capacity stratified by sex is presented in Fig. 4. As expected, the limits of agreement were wider than in the developed model. The limit of agreement for males was − 8.59 mL·L^− 1^ to 7.76 mL·L^− 1^. For females, the limit of agreement ranged between − 9.21 mL·L^− 1^ to 9.87 mL·L^− 1^.

## Discussion

To the best of our knowledge, this is the first external validation of prediction equations for OUEP. Moreover, there is no research so far that has evaluated the stability of OUEP in EA. In this article we found that following areas: (1) OUEP is a replicable cardiorespiratory measure between EA and untrained healthy individuals, (2) OUEP predicted by somatic measurements provided promising but limited accuracy and (3) OUEP is a valuable marker when stratifying cardiorespiratory response profiles in EA.

EA is a unique population. They do not fit into the general reference values for the majority of cardiovascular measurements [33]. Regular physical activity has a brilliant preserving effect on the cardiovascular system. Endurance training causes a slower decline of fitness with aging (e.g. VO_2_peak or maximal heart rate) [8, 34]. Our previous studies showed significant inaccuracies in prediction models for VO_2_peak or heart rate when applied to athletic individuals [20, 21]. So far as we know, OUEP was never deeply analyzed in context of sports cardiology in EA.

OUEP remains a relatively understudied marker. Sun et al. found that, when OUEP drops below 65% of the predicted value, it can suggest pathology [19]. In our EA, no one observed such a difference. Only two females showed a difference of 11.3 mL·L^− 1^. For all the remaining participants calculated OUEP did not differ more than 10 mL·L^− 1^, i.e. around 25% error. What is more, no one exceeded the 35%. The highest calculated underestimation was 27.7% (11.3 mL·L^− 1^ in both females). The variance explained by the derived model was 12.9% (R^2^ = 0.129). Comparably, the variance explained by the external model [17] was 9.9% (R^2^ = 0.099). Although those values are limited, both are comparable, even though the models were developed from different populations.

It is worth noting that the only variable missing from the original equation presented by Sun et al. is age [17]. This probably results from our cohort age distribution (22.7 ± 4.6 years old). Age had only minor variance in our homogenous sample and did not reach significance. This is an emerging point in the discussion of our results and a great recommendation for further studies on OUEP predictions in populations of EA with a wider age distribution. Moreover, the original derivation study by Sun et al. included only 57 well-trained participants [17]. Our study has a wider population of 140 EA. Therefore, the provided results seem to be more reliable.

Moreover, OUEP has further advantages. Assuming other measures of cardiorespiratory fitness, VO_2_peak may be different if a verification retest is used and the ventilatory efficiency slope depends largely on the plotting method [35, 36]. OUEP is an objective measure because it is an averaged time interval; thus, OUEP seems reliable and should be easier to compare between studies [17, 19]. An interesting finding from our study is presented on the Figs. 3 and 4 where bias grow simultaneously with increasing OUEP [37]. This indicate that agreement between measured and estimated OUEP could not be constant but varies with fitness level. Perhaps, prediction of OUEP with a universal equation could not be the most valid method. Therefore, it is justified to derive the models tailored for particular populations (i.e. trained and untrained) [8, 37].

The relationship of OUEP to basic demographic parameters such as sex and age remained mostly similar between EA and the general population [17]. However, findings from our study should be discussed as some relationships to other demographic measures with OUEP seem to be complex. We highlight the very strong impact of sex as reflected by the high β-coefficient (β = 0.54). As expected, males had higher OUEP than females. Even though our study population was younger (age approximately 22.3 ± 4.6 years), OUEP also declined with increasing age (β= − 0.048, *p* = 0.57). This relationship was not significant; thus, we do not include age covariate in our models. Our model indicates that OUEP could decrease with height (β=–0.257, *p* = 0.041). Finally, we did not find a significant relationship between OUEP and other somatic measurements such as body weight, BMI, or BSA.

In young people, maximal effort is strongly dependent on the motivation to continue effort despite fatigue [38]. This does not mean that if CPET was submaximal, it did not provide valuable data. OUEP is most often found close to the first ventilatory threshold [17]. Our study indicates that it is a robust cardiorespiratory verifier, no matter whether the maximum effort has been achieved or not. Since OUEP is measured during submaximal, not peak, exercise, It does not cause strenuous fatigue and is safer for clinical purposes or to avoid overtraining [39]. OUEP can be repeated frequently and regularly to monitor cardiorespiratory health. What is more, OUEP is easier to reliably determine because it is calculated from a time interval and does not include finding the ventilatory threshold which could be affected by interobserver variability [40, 41].

### Limitations

To ensure that our conclusions will be correctly interpreted, some points should be raised. We gathered a population of high-performance well-trained individuals, which is difficult. According to Wasserman & Hansen, the predicted VO_2_peak in our subjects was on average 144.5 ± 25.9% [42]. Therefore, we were able to conduct external validation on EA. Participants with VO_2_peak > 140% predicted were assigned to ‘very fit’ in the original study by Sun et al. [17]. We also emphasize the equal ratio of males to females (55–45%). Therefore, the influence of sex is balanced and reliably modelled.

The study group appears to be homogeneous, and in the majority consisted of younger Caucasian EA [43]. Further studies with a wider age distribution should be conducted. Those studies should include both pediatric EA and master EA. CPET was conducted in a cycling protocol. The modality of CPET could influence the results. In other protocols (e.g. running or rowing) there could be some different values [44]. In the original derivation study from Sun et al. the participants also underwent CPET on the treadmill [17]. CPET results are usually slightly higher during running than cycling [44]. We stipulate that this should not have wide impact on results of our study because OUEP is a ratio of two variables. However, this relationship should be verified by further researchers. Therefore, we highlight that derived models are specified for cycling CPET.

It is worth noting that there is some ambiguity of the maximum effort criteria used for studies on EA. Wagener et al. suggest that the most appropriate RER for individuals aged 20–39 is ≥ 1.13 [45]. On the other hand, the American Thoracic Society and with American College of Chest Physicians recommend a RER of 1.10 [4]. Finally, Petek et al. found that the most suitable RER for EA equals 1.05 [8]. In our study, we used RER of 1.05 as a cutoff (i.e. similar to Petek et al.) because these criteria are the latest ones and were provided after consideration and evaluation of previous reports [8]. However, if any future study will choose other criteria of maximal CPET, we underline that the OUEP results could be slightly different. We underline the need to derive further models to predict OUEP from other testing modalities and under other testing criteria. In summary, all the results should be extrapolated carefully.

### Clinical implications

Some practical and clinical applications should also be discussed. CPET can be performed for sports diagnostics to guide training and in a clinical setting when pathology is suspected [46, 47]. However, clinicians need a certain reference point (i.e., a value or formula to compare with directly measured results). Retrospective evaluation of achieved OUEP could facilitate the assessment of cardiorespiratory fitness [17]. Furthermore, the prospective calculation of OUEP enables the setting of the target ranges when planning the CPET intensity [17]. Our prediction equations are a valuable part of a clinician’s toolbox when assessing cardiorespiratory health. However, the provided equations should not be used to make a definitive diagnosis. Nevertheless, the models could be used to guide further steps. This study facilitates the implementation of OUEP among apparently healthy subjects and those with suspected CVD.

### Future perspectives

Previous studies tested OUEP in predicting the VO_2_peak [38, 48]. Most often there was a weak or limited correlation between OUEP and VO_2_peak (17, 50). However, in our study, both parameters were significantly correlated (*R* = 0.32, *p* < 0.001). OUEP and VO_2_peak describe different elements of exercise physiology and complement each other but do not replace one another [17, 19]. Future research on wider populations should clarify how OUEP links with VO_2_peak. OUEP certainly constitutes an interesting supplement to VO_2_peak [48]. OUEP is emerging as an interesting additional cardiorespiratory variable [19]. Future studies should verify whether OUEP remains stable in extreme age groups. Therefore, further research about OUEP could be conducted on junior and master EA. Another unanswered point is whether OUEP has any discriminative power when CVD is suspected in EA or could help to identify athletes in a state of overtraining.

## Conclusions

OUEP remains stable and is only minimally influenced by endurance level. It is transferable between untrained individuals and EA. OUEP could be modelled in EA with basic demographic parameters: height and sex. Prediction equations for OUEP were replicable and provided promising, however limited accuracy. Medical professionals and fitness practitioners should consider OUEP when evaluating CPET results to determine cardiorespiratory fitness and monitor training.

### Electronic supplementary material

Below is the link to the electronic supplementary material.

Supplementary Material 1

Supplementary Material 2

## Data Availability

The data were be made available for a reasonable request to the corresponding author.

## Abbreviations

BSA: Body surface area
CPET: Cardiopulmonary exercise test
CVD: Cardiovascular diseases
EA: Endurance athletes
OUEP: Oxygen uptake efficiency plateau
OUES: Oxygen uptake efficiency slope
R: Pearson correlation coefficient
R^2^: Coefficient of determination
RER: Respiratory exchange ratio
RMSE: Root-mean-square error
VCO_2_: Carbon dioxide output
VE: Minute ventilation
VO_2_: Oxygen uptake
VO_2_ Peak: Peak oxygen uptake
