# Supplementary material for: Oxygen uptake efficiency plateau is unaffected by fitness level - the NOODLE study
Source: BMC Sports Sci Med Rehabil. 2024 Jul 10;16:151. doi: 10.1186/s13102-024-00939-w (PMC11234747; doi:10.1186/s13102-024-00939-w)
Supplement: Supplementary file 2 — Supplementary Material 2 [file 13102_2024_939_MOESM2_ESM.pdf]

SUPPLEMENTARY MATERIAL 2

Table S2. Participants demographic and exercise characteristics.

Variable	Total population [*N*=140]	Males [*N*=77]	Females [*N*=63]
**Age (years)**	22.7 ± 4.6	21.8 ± 4.8	23.8 ± 4.2
**Height (cm)**	174.8 ± 9.9	181.6 ± 6.3	166.3 ± 6.2
**Weight (kg)**	69.3 ± 10.1	76.1 ± 7.6	61.0 ± 5.5
**BMI ($kg·m^{-2}$)**	22.6 ± 1.7	23.1 ± 1.7	22.1 ± 1.6
**HR ($beats·min^{-1}$)**	190.9 ± 8.9	190.8 ± 8.7	191.0 ± 9.1
**VE ($L·min^{-1}$)**	154.5 ± 34.1	176.3 ± 26.3	127.8 ± 21.1
**VO_2peak ($L·min^{-1}$)**	3.86 ± 0.82	4.40 ± 0.64	3.21 ± 0.48
**VO_2peak/kg ($mL·kg^{-1}min^{-1}$)**	55.2 ± 8.6	57.8 ± 9.0	52.1 ± 7.0
**% pred. VO_2peak**	144.5 ± 25.9	161.4 ± 21.8	130.6 ± 20.2
**VCO_2 ($L·min^{-1}$)**	4.36 ± 0.96	5.00 ± 0.72	3.57 ± 0.52
**RR ($breaths·min^{-1}$)**	60.0 ± 7.6	59.9 ± 8.3	60.2 ± 6.7
**VT (L)**	2.81 ± 0.64	3.22 ± 0.53	2.30 ± 0.32
**RER (VO_2/VCO_2)**	1.14 ± 0.05	1.15 ± 0.05	1.13 ± 0.05
**O_2P (VO_2/HR)**	20.7 ± 4.4	23.5 ± 3.3	17.3 ± 3.0
**Ramp time (minutes)**	21.3 ± 2.6	21.4 ± 2.6	21.1 ± 2.7
**Workload (watts)**	320.4 ± 76.2	364.4 ± 70.0	266.7 ± 40.8
**OUES ($mL·min^{-1}/L·min^{-1}$)**	3.96 ± 0.90	4.41 ± 0.87	3.41 ± 0.58
**OUEP ($mL·L^{-1}$)**	42.7 ± 4.7	44.2 ± 4.2	41.0 ± 4.8

Abbreviations: BMI, body mass index; HR, peak heart rate; VE, peak minute ventilation; VO_2peak, peak oxygen uptake; VCO_2, peak carbon dioxide output; RR, peak respiratory rate; VT, tidal volume; RER, peak respiratory exchange ratio; O_2P, peak oxygen pulse; OUES, oxygen uptake efficiency slope; OUEP, oxygen uptake efficiency plateau.

Note: Measures are presented as: mean ± standard deviation. RER was calculated from VO_2/VCO_2 from the same 15-sec interval at the end of exercises. O_2P was calculated from VO_2/HR in the same 15-sec period. OUES was calculated from VO_2/VE_{log} during whole exercise effort. OUEP was considered as the highest continuous 90-sec average from the VO_2/VE ratio. Predicted VO_2peak was calculated from Wassermann & Hansen equation.
